# Insights on the performance of phenotypic tests versus genotypic tests for the detection of carbapenemase-producing Gram-negative bacilli in resource-limited settings

**DOI:** 10.1186/s12866-022-02660-5

**Published:** 2022-10-14

**Authors:** Noha A. Kamel, Sally T. Tohamy, Ibrahim S. Yahia, Khaled M. Aboshanab

**Affiliations:** 1 Department of Microbiology, Faculty of Pharmacy, Misr International University (MIU), 19648 Cairo, Egypt; 2 Department of Microbiology & Immunology, Faculty of pharmacy-Girls, Al-Azhar University, 11651 Cairo, Egypt; 3 Laboratory of Nano-Smart Materials for Science and Technology (LNSMST), Department of Physics, Faculty of Science, and Research Center for Advanced Materials Science (RCAMS), King Khalid University, P.O. Box 9004, 61413 Abha, Saudi Arabia; 4 Nanoscience Laboratory for Environmental and Biomedical Applications (NLEBA), Semiconductor Lab, Department of Physics, Faculty of Education, Ain Shams University, 11757 Roxy, Cairo Egypt; 5 Microbiology and Immunology Department, Faculty of Pharmacy, Ain Shams University, African union organization Street, 11566 Abbassia, Cairo Egypt

## Abstract

**Background:**

Carbapenemase-producing Gram-negative (CPGN) bacteria impose life-threatening infections with limited treatment options. Rigor and rapid detection of CPGN-associated infections is usually associated with proper treatment and better disease prognosis. Accordingly, this study aimed at evaluating the phenotypic methods versus genotypic methods used for the detection of such pathogens and determining their sensitivity/specificity values.

**Methods:**

A total of 71 CPGN bacilli (30 *Enterobacterales* and 41 non-glucose-fermenting bacilli) were tested for the carbapenemase production by the major phenotypic approaches including, the modified Hodge test (MHT), modified carbapenem inactivation method (mCIM), combined disk test by EDTA (CDT) and blue-carba test (BCT). The obtained results were statistically analyzed and correlated to the obtained resistant genotypes that were determined by using polymerase chain reactions (PCR) for the detection of the major carbapenemase-encoding genes covering the three classes (Class A, B, and D) of carbapenemases.

**Results:**

In comparison to PCR, the overall sensitivity/specificity values for detection of carbapenemase-producing organism were 65.62%/100% for MHT, 68.65%/100% for mCIM, 55.22%/100% for CDT and 89.55%/75% for BCT. The sensitivity/specificity values for carbapenemase-producing *Enterobacterales* were, 74%100% for MHT, 51.72%/ 100% for mCIM, 62.07%/100% for CDT and 82.75%/100% for BCT. The sensitivity/specificity values for carbapenemase-producing non-glucose fermenting bacilli were, 62.16%/100% for MHT, 81.57%/100% for mCIM, 50/100% for CDT and 94.74%/66.66% for BCT. Considering these findings, BCT possess a relatively high performance for the efficient and rapid detection of carbapenemase producing isolates. Statistical analysis showed significant association (*p <* 0.05) between *bla*_NDM_ and/or *bla*_VIM_ genotypes with MHT/CDT; *bla*_KPC_/*bla*_GIM_ genotypes with CDT and *bla*_GIM_ genotype with BCT.

**Conclusion:**

The current study provides an update on the performance of the phenotypic tests which are varied depending on the tested bacterial genera and the type of the carbapenemase. The overall sensitivity/specificity values for detection of CPO were 65.62%/100% for MHT, 68.65%/100% for mCIM, 55.22%/100% for CDT and 89.55%/75% for BCT. Based on its respective diagnostic efficiency and rapid turnaround time, BCT is more likely to be recommended in a resource-limited settings particularly, when molecular tests are not available.

**Supplementary Information:**

The online version contains supplementary material available at 10.1186/s12866-022-02660-5.

## Background

Carbapenemases are β-lactamases produced by carbapenem-resistant (CR) bacteria as their major antimicrobial-resistance mechanisms [1, 2]. Owing to their broad spectrum of activity and stability towards the majority of β-lactamases, carbapenems are considered one of the last lines of therapy to treat severe infections caused by multi-drug resistant (MDR) bacteria [2]. However, the emergence of CR among clinically relevant Gram-negative bacilli (GNB) as *Enterobacterales* (such as *E. coli, K. pneumoniae*) and non-glucose fermenting bacilli (such as *A. baumannii, Pseudomonas aeruginosa*) has become globally recognized as one of the most serious challenges to clinicians in dealing with community-acquired and health-care-associated infections [3, 4].

Generally, mechanisms contributing to CR could be through overexpression of efflux pump, decrease in outer membrane permeability coupled with hyper-production of AmpC β-lactamases, and finally through the production of carbapenemase enzymes as the most predominant resistance mechanism [1, 5]. Carbapenemases are β-lactamases that belong to different Ambler classes (A, B, and D). Class A (KPC) and class D (OXA) are serine carbapenemases that depends on serine as an enzyme active center while, class B (NDM, IMP, GIM, VIM) are metallo-β-lactamase enzymes that require Zinc for their activity [6–8]. By far infections caused by carbapenemase-producing organisms (CPOs) either *Enterobacterales* (CPE) or non-glucose fermenting bacilli (CP-NF) are associated with higher mortality rates ranging from 40 to 50% [9] and widespread dissemination as the majority of carbapenemase genes are carried on mobile genetic elements [10], compared to non-CPOs. The CPOs are also resistant to commonly used antibiotics such as aminoglycosides, fluoroquinolones and tetracyclines that will consequently reduce therapeutic options and prolong hospitalization [11, 12]. Accordingly, a variety of laboratory methods is urgently required to allow prompt screening, identification, and implantation of appropriate infection control measures to limit spread of these difficult to treat CPOs.

Despite of being the gold standard in identification of well-known carbapenemase encoding genes, molecular tests are often expensive and require specialized staff, rendering these techniques unavailable for routine clinical practice especially in resource limited settings [13]. To overcome these challenges, several simple and affordable phenotypic tests were recommended by clinical and laboratory standard institute (CLSI) that allow effective detection of CPOs particularly, CPGN pathogens. Currently, growth-based assays and colorimetric hydrolysis methods (blue carba test: BCT, Carba Nordmann/ Poirel: Carb NP) that depends on the growth of the organism in presence of carbapenem antibiotics and degradation of carbapenem products, respectively are commonly used in clinical practice [1, 7, 14]. Of note, CLSI had archived MHT, while other methods as mCIM and Carba NP were endorsed.

Unsuccessful and slow in the diagnosis of CPGN-associated infections is complicated by the treatment failure and patient death. These urges evaluating and regular updates on the sensitivity/specificity values of the existed phenotypic and genotypic diagnostic methods of the most common circulating CPGN pathogens. Although, the phenotypic detection and molecular characterization of CPGN pathogens had been previously reported in literature [13–16], still more studies are urgently required to validate the laboratory performance (sensitivity/specificity/positive predictive value/negative predictive value) of the respective phenotypic methods and to correlate them with the different types of carbapenemase enzymes [1, 14]. Therefore, in this study we aimed to evaluate the performance of four major phenotypic approaches namely MHT, mCIM, BCT, combined disk test by EDTA (CDT) for detecting CPGN pathogens. This was followed by PCR detection of the mostly abundant carbapenemases for exploring the correlation between the associated genotypes and phenotypic tests. The obtained findings of this research will be of a particular importance for determining the most convenient method(s) for rapid and accurate detection of CPGN pathogens particularly, for laboratories with resource-limited settings.

## Materials and methods

### Bacterial isolates and identification

Over a period of 12 months (January 2021-December 2021), a total of 71 GNB isolates with reduced susceptibility to carbapenem (ertapenem, imipenem and meropenem) by disk diffusion method were collected from the microbiology laboratories of El-Demerdash a tertiary care hospital with 3200 beds, Cairo, Egypt [17]. This study was conducted in accordance with the Declaration of Helsinki, reviewed and approved by the Faculty of Pharmacy, Ain Shams University Research ethics committee, (ACUC-FP-ASU RHDIRB2020110301 REC #72).

The respective isolates were recovered from unidentified clinical specimens including urine (a midstream urine specimen was collected from patients suffered from urinary tract infection), blood (In case of blood stream infections) and sputum (In case of pneumonia) as a routine care of patients admitted to the hospital. Based on the hospital guidelines, an informed consent was obtained from all patients and/or their legal guardian(s) after clarifying to them the objective of this study. For preliminary isolation of GNB, a set of nutrient agar, blood agar, chocolate agar and MacConkey agar were used. For subsequent studies, isolates were preserved at -80 °C. Identification of collected clinical isolates was checked based on microscopic, macroscopic and conventional biochemical tests as mentioned in Bergey’s manual of determinative bacteriology [18]. Briefly, macroscopic evaluation involves description of size, shape, elevation, texture, margin and optical character of colonies. A set of biochemical tests including oxidase test, sugar fermentation, triple sugar iron, indole production/ methyl red / Voges Proskauer and citrate utilization (IMVC test) were used to identify Gram negative bacteria. The identification of the recovered isolates was confirmed using the automated microbial identification system, Vitek-2 system (bioMérieux, Marcy L’Etoile, France).

### Antimicrobial susceptibility tests

The antibiotic susceptibility testing was determined by Kirby-Bauer disk diffusion method and a panel of 19 antibiotics disks including amoxicillin/clavulanic acid (AMC: 20 µg/10µg), ampicillin (AM:10 µg), amikacin (AK: 30 µg), aztreonam (AZM: 30 µg), cefotaxime (CTX: 30 µg), ciprofloxacin (CIP: 5 µg), ceftriaxone (CRO: 30 µg), ceftazidime (CAZ: 30 µg), cefepime (FEP: 30 µg), cefoxitin (FOX: 10 µg), colistin (CT: 10 µg), ertapenem (ETP: 10 µg), gentamicin (CN: 10 µg), imipenem (IPM: 10 µg), levofloxacin (LEV: 10 µg), meropenem (MEM: 10 µg), ampicillin/ sulbactam (SAM: 10/10 µg), trimethoprim/sulfamethoxazole (SXT: 1.25/23.75ug) and tigecycline (TGC: 15 µg) obtained from Oxoid, Basingstoke, United Kingdom were tested. According to the CLSI guidelines [17], isolates that showed resistance to at least one of the above-mentioned carbapenems were considered CR- GNB while, isolates that were not susceptible to at least one agent in three or more antimicrobial categories were recorded as MDR- GNB [19]. To confirm CR, minimum inhibitory concentration (MIC) of meropenem was determined by broth microdilution method. Isolates that tested intermediate or resistant to current CLSI breakpoints of meropenem (MIC ≥ 2–4 µg/ml for CPE and ≥ 4–8 µg/ml for CP-NF) were considered as potential carbapenemase producers [16]. A flow chart for detection of CPO was shown in supplementary file Fig S1.

### Phenotypic tests for detection of carbapenemase producing organisms (CPO)

#### -Modified Hodge test (MHT)

An overnight culture of *E. coli* ATCC 25,922 (quality control/indicator strain) equivalent to 0.5 McFarland was diluted 1:10 using sterile saline solution and thereafter was streaked as a lawn on a Mueller Hinton agar plate. After placing meropenem or ertapenem disk at center of plate, 3 ± 5 colonies of overnight tested isolates were streaked from edge to the central disk using sterile swabs and thereafter plates were incubated at 37 °C for 24 h in ambient of air. The production of carbapenemase enzymes were indicated by the appearance of clover leaf like indentation and enhanced growth of indicator *E. coli* strains [20]. Phenotypic tests namely MHT, mCIM, BCT and CDT were done in duplicate to ensure reproducibility.

#### -Modified carbapenem inactivation method (mCIM)

An overnight culture of tested bacteria (1 µl loopful for *Enterobacterales* and 10 µl for non-glucose fermenting GNB including, *P. aeruginosa*, *A. baumannii* and *Stenotrophomonas maltophilia*) was suspended in 2ml trypticase soy broth (TSB). Meropenem disk was added to the vortexed suspension and broth was incubated for 4 h at 37 °C. Just prior to completion of 4 h incubation, an overnight culture of *E. coli* ATCC 25,922 (adjusted to 0.5 McFarland), was streaked over the surface of Mueller-Hinton agar plate and meropenem disk was introduced to center of plate. Plates were incubated in ambient air at 37 °C for 18 to 24 h. Tested isolates that showed an inhibition zone between 6 and 15 mm or presence of pinpoint colonies within 16–18 mm zone were considered CPO. Tested isolates that showed an inhibition zone ≥ 19 mm were considered carbapenemase negative [20].

#### -Blue-carba test (BCT)

The BCT is a rapid biochemical test that depends on in vitro hydrolysis of imipenem and release of an acid that can be detected by bromothymol blue indicator. In a microtiter plate, a loopful of tested bacterial culture was added to both tested solution (0.04% bromothymol blue, 0.1mmol/liter ZnSO4 and 3 mg/ml of imipenem with a final pH adjusted to 7) and negative control solution (0.04% bromothymol blue adjusted to pH = 7). After about 2 h, the presence of CPO caused a shift in the pH and turned the tested solution into either yellow or green color, while non-CPO remained blue in color [21–23].

#### -Combined disk test (CDT)

A Mueller-Hinton agar was over-streaked by an overnight culture of the tested bacterium adjusted to 0.5 McFarland. Imipenem and imipenem/ EDTA disks (Oxoid, Basingstoke, United Kingdom) were placed on the surface of the agar and thereafter plates were incubated at 37 °C for 24 h. Production of class B carbapenemase was indicated by enhancement of inhibition zone ≥ 5 mm for latter disk when compared to former disk [24].

### Molecular characterization of carbapenemase producing GNB

Overnight cultures of tested isolates were grown in Luria Bertani (LB) broth containing 25 µg/mL meropenem. PCR detection and conditions of carbapenemase-encoding genes was carried out using PCR as previously described [25]. The set of 5 primers with the corresponding annealing temperatures and expected amplicon sizes was shown in supplementary file (Table S1). The molecular characterization tests were performed from the colonies of the same plate where phenotypic tests were performed whenever possible to ensure uniformity, reproducibility and effective comparison of the results.

**Table 1:** Evaluation of sensitivity, specificity, positive predictive value and negative predictive value of phenotypic tests

Test	GNB	TP^a^	FP^b^	FN^c^	TN^d^	Sensitivity	Specificity	PPV	NPV
95% confidence interval [95% CI]									
MHT	*Enterobacterales* (n = 28)	20	0	7	1	74% [53.40-88.12]	100% [5.46–100]	100% [79.95–100]	12.50% [0-53.32]
	Non glucose fermenting bacilli (n = 40) *A. baumannii* *P. aeruginosa*	23 16 6	0 0 0	14 8 0	3 2 1	62.16% [44.78–77.06] 66.66% [44.69–83.57] 50% [22.28–77.71]	100% [31–100] 100% [19.78–100] 100% [5.46–100]	100% [82.19–100] 100% [75.92–100] 100% [51.68–100]	17.64% [4.67–44.19] 20% [3.54–55.78] 14.28% [0.78-58]
	total tested = 68	43	0	21	4	65.62% [52.61–76.75]	100% [39.57–100]	100% [89.56–100]	15.38% [5.04–35.72]
m-CIM	*Enterobacterales* (n = 30)	15	0	14	1	51.72% [32.89–70.10]	100% [5.46–100]	100% [74.65–100]	6.66% [3.49–33.96]
	Non glucose fermenting bacilli (n = 41) *A. baumannii* *P. aeruginosa*	31 19 11	0 0 0	7 6 1	3 2 1	81.57% [65.10-91.67] 76% [49.65–85.50] 91.66% [62.08–99.60]	100% [31–100] 100% [19.78–100] 100% [5.46–100]	100% [86.27–100] 100% [79.07–100] 100% [67.85–100]	30% [8.09–64.63] 25% [4.45–64.42] 50% [2.66–97.33]
	total tested = 71	46	0	21	4	68.65%, [56.02–79.13]	100%, [39.57–100]	100%, [90.39–100]	16%, [5.25–36.91]
BCT	*Enterobacterales* (n = 30)	24	0	5	1	82.75% [63.51–93.47]	100% [5.46–100]	100% [82.82–100]	16.66% [1.05–70.12]
	Non glucose fermenting bacilli (n = 41) *A. baumannii* *P. aeruginosa*	36 23 12	1 0 1	2 2 0	2 2 0	94.74% [80.93–99.08] 92.56% [72.49–98.60] 100% [69.87–100]	66.66% [12.53–98.23] 100% [19.78–100] 0% [0-94.53]	97.29% [84.19–99.85] 100% [82.19–100] 92.30% [62.08–99.59]	50% [9.18–90.81] 50% [9.18–90.81] NA
	total tested = 71	60	1	7	3	89.55% [79.06–95.34]	75% [21.94–98.68]	98.36% [90.01–99.91]	30% [8.09–64.63]
CDT	*Enterobacterales* (n = 30)	18	0	11	1	62.07 [42.36–78.69]	100% [5.46–100]	100% [78.12–100]	8.33% [0-40.24]
	Non glucose fermenting bacilli (n = 41) *A. baumannii* *P. aeruginosa*	19 15 4	0 0 0	19 10 8	3 2 1	50% [33.65–66.34] 60% [38.89–78.18] 33.33% [11.27–64.56]	100% [31–100] 100% [19.78–100] 100% [5.46–100]	100% [79.07–100] 100% [74.65–100] 100% [39.57–100]	13.63% [3.58–35.96] 16.66% [2.94–49.11] 11.11% [0.58–49.32]
	total tested = 71	37	0	30	4	55.22%, [42.63–67.21]	100%, [39.57–100]	100%, [88.28–100]	11.76% [3.83–28.39]

TP^**a**^, true positive, FP^**b**^, False positive, FN^**c**^, False negative, TN^**d**^, true negative; Sensitivity = a/(a + c), Specificity = d/(b + d), PPV (Positive predictive value) = a/(a + b), NPV (Negative predictive value) = d/ (c + d); MHT, modified Hodge test, mCIM, modified carbapenem inactivation method, CDT, combined disk test by EDTA ; BCT, blue-carba test

* Non glucose fermenting included *A. baumannii, P. aeruginosa* and *Stenotrophomonas maltophilia* isolates

### Phenotypic analysis using heatmap signature

Morpheus online software (https://software.broadinstitute.org/morpheus/ accessed on 25 March 2022) was used to generate a dendrogram showing heatmap signatures of the isolates, to determine their phenotypic relatedness based on the antimicrobial resistance pattern and the production of carbapenemase enzymes as determined by MHT, mCIM, BCT, CDT by EDTA and PCR. An excel file of required data including isolate code, antibiotic sensitivity, results of 4 phenotypic tests and carbapenemase genes was uploaded and then the interactive tools of Morpheus was used to create hierarchical clustering, annotations and display chart.

### Statistical analysis

The sensitivity, specificity, positive predictive value (PPV) and negative predictive value (NPV) of each phenotypic test along with their 95% confidence interval (CI) were calculated using the free software VassarStats (http://vassarstats.net/; accessed on 1 February 2022). The clinical research calculator namely calculator 1 was used to determine the previously mentioned values along with 95% CI. Pearson’s chi-square test was used to analyze the association of different types of carbapenemase enzymes with each phenotypic test. Spearman’s correlation was performed to determine the strength between all possible phenotypes and genotypes combinations. The strength of Spearman’s correlation coefficient varies between + 1 and − 1 value. Values between 0 ± 0.3, 0.4 ± 0.6 and 0.7 ± 1 indicates a weak, moderate strength and strong relationship, respectively. Minitab version 19 was used for statistical analysis, *P*-value < 0.05 were considered statistically significant results.

## Results

### Overview on the carbapenem-resistance Gram-negative bacilli (CR-GNB) isolates and antimicrobial susceptibility testing

Of 71 CR-GNB included in the study, 32 (45%), 22 (30.9%), and 17 (23.9%) were derived from sputum, blood, and urine specimens, respectively.

**Fig. 1:**
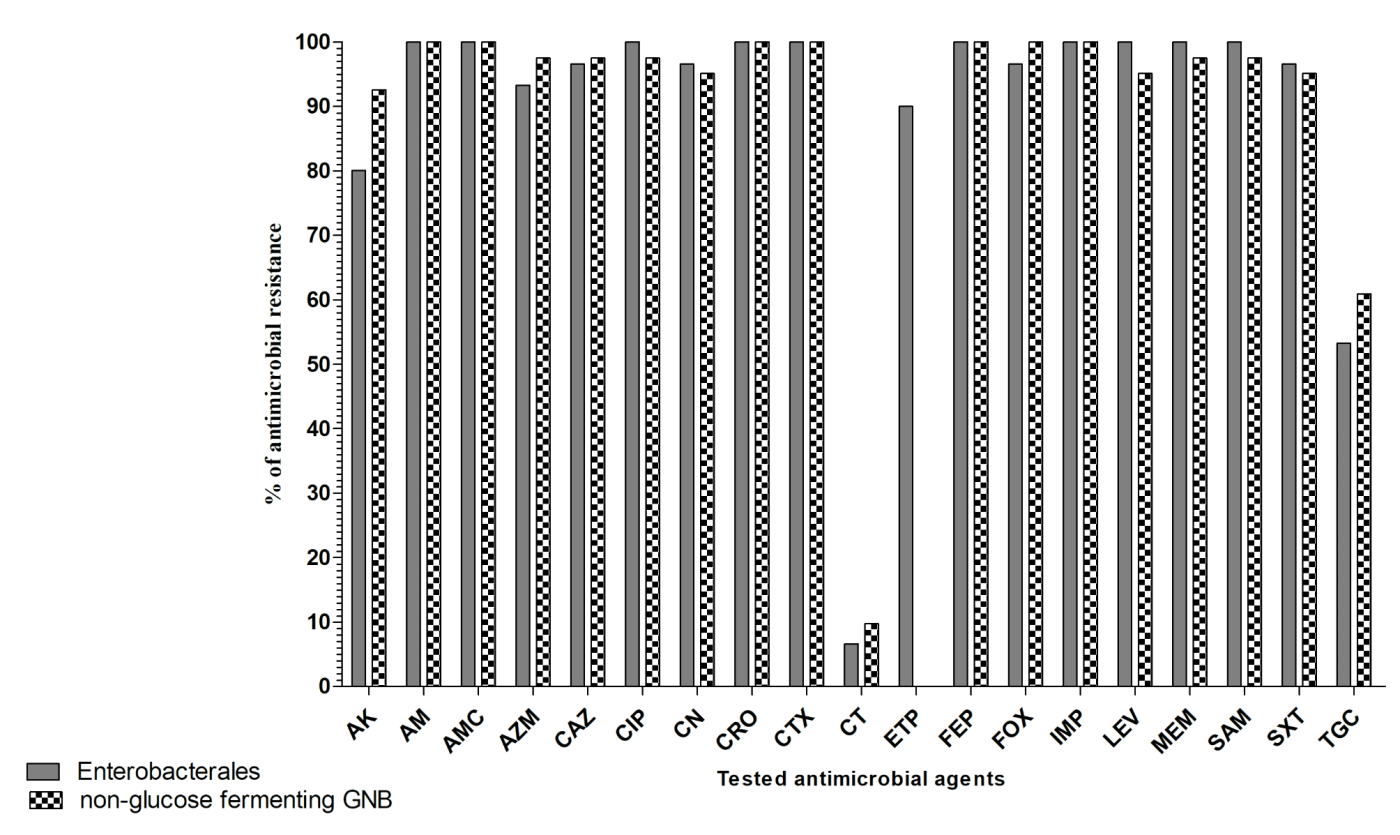
The antibiogram analysis of Carbapenemase-producing Gram-negative (CPGN) pathogens (n = 71) including, *Enterobacterales* and non-glucose-fermenting Gram-negative bacilli (GNB) against various classes of antimicrobial agents

The antibiogram analysis of CR-GNB against various classes of antimicrobial agents including β-lactam group (AMC, AM, AZM, CTX, CRO, CAZ, FEP, FOX, IPM, MEM, SAM, ETP), aminoglycosides (AK, CN), quinolones (CIP, LEV), polymyxins (CT), glycylcyclines (TGC) and sulfonamides/diaminopyrimidines (SXT) was depicted in Fig. 1. The results revealed that the antimicrobial resistance profile of the tested CRE and CR-NF had exceeded 80% for all β-lactam group, aminoglycosides, quinolones and sulfonamides/diaminopyrimidines. On the other hand, the highest susceptibility profile was recorded towards CT ranging from 90.3 to 93.4% and followed by TGC ranging from 39.1 to 46.7%. About 98.6% (70/71) of the tested isolates had shown acquired resistance to at least one agent in three or more antimicrobial classes and were considered MDR. Overall, 95.77% (68/71) of the tested CR-GNB isolates were not susceptible to meropenem MIC and were defined as potential CPO.

**Fig. 2:**
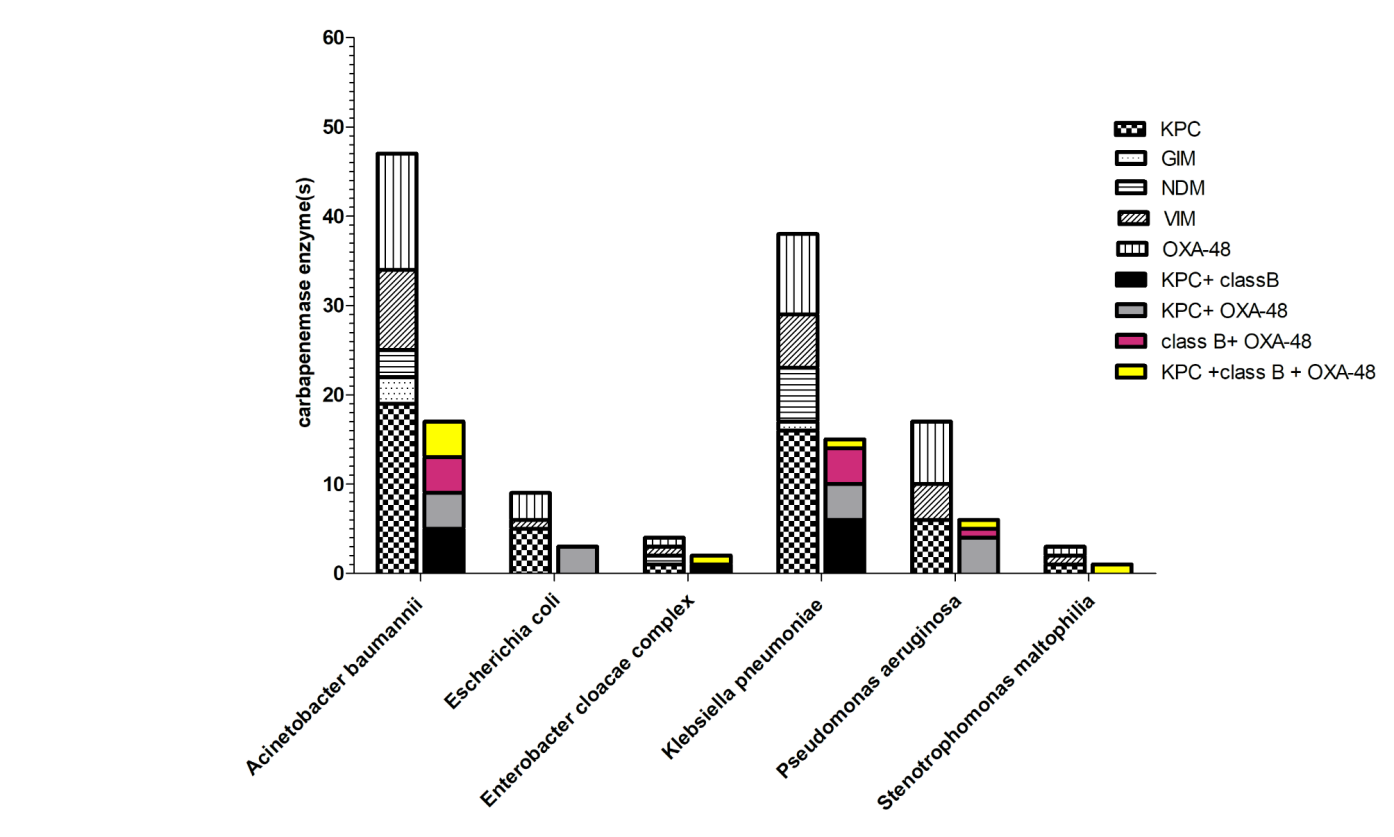
Prevalence of different carbapenemase genotypes among the recovered carbapenem-resistance Gram-negative bacilli (CR-GNB; n = 71).). *Bla*_KPC_, *gene coded for Klebsiella pneumoniae* carbapenemases (KPC); *bla*_NDM_, a gene coded for New Delhi metallo-β-lactamase (NDM); imipenem-resistant *Pseudomonas*-type carbapenemases (IMP); *bla*_VIM_, a gene coded for Verona integron-encoded metallo-β-lactamase (VIM); *bla*_GIM_, a gene coded for German imipenemase and *bla*_OXA−48,_ oxacillinase (OXA-48-like) types

Out of 71 CR-GNB isolates, 67 (94.36%) were CPO. Of these carbapenemases, *bla*_KPC_ was the most frequent (49, 73.13%), followed by *bla*_OXA−48_ (35, 52.23%), *bla*_VIM_ (23, 34.32%), *bla*_NDM_ (10, 14.92%) and *bla*_GIM_ (4, 5.97%) as depicted in Fig. 2. Out of 67 CPO isolates, 44 (65.67%) produced more than one carbapenemase genes. Co-existence of *bla*_KPC_ and *bla*_OXA−48_ was the most dominant (34%), followed by co-detection of class A and class B (27.2%), then class B and class D (20.4%) and finally 3 classes (18.1%). The 4 non-CPO isolates comprised 2 isolates of *A. baumannii* and 1 isolate of *K. pneumoniae* and *P. aeruginosa*.

### Performance of phenotypic tests for detection of CPO

The performance of various phenotypic assays (MHT, mCIM, BCT and CDT by EDTA) for detection of CPO were shown in Table 1. Growth based method as MHT and mCIM had shown a sensitivity of 65.62% and 68.65%, respectively for detection of CPO while, both tests had shown 100% specificity. The BCT had a sensitivity of 89.55% and specificity of 75% while; CDT by EDTA had a sensitivity of 55.22% and specificity of 100%. For CPE, the sensitivity, specificity, PPV and NPV of four phenotypic tests was 51-82.75%, 100%, 100% and 6.66%-16.66%, respectively. For CP-NF, the sensitivity, specificity, PPV and NPV of four phenotypic tests had ranged from 50 to 94.74%, 66-100%, 97.29-100% and 13.63-50%, respectively.

**Fig. 3:**
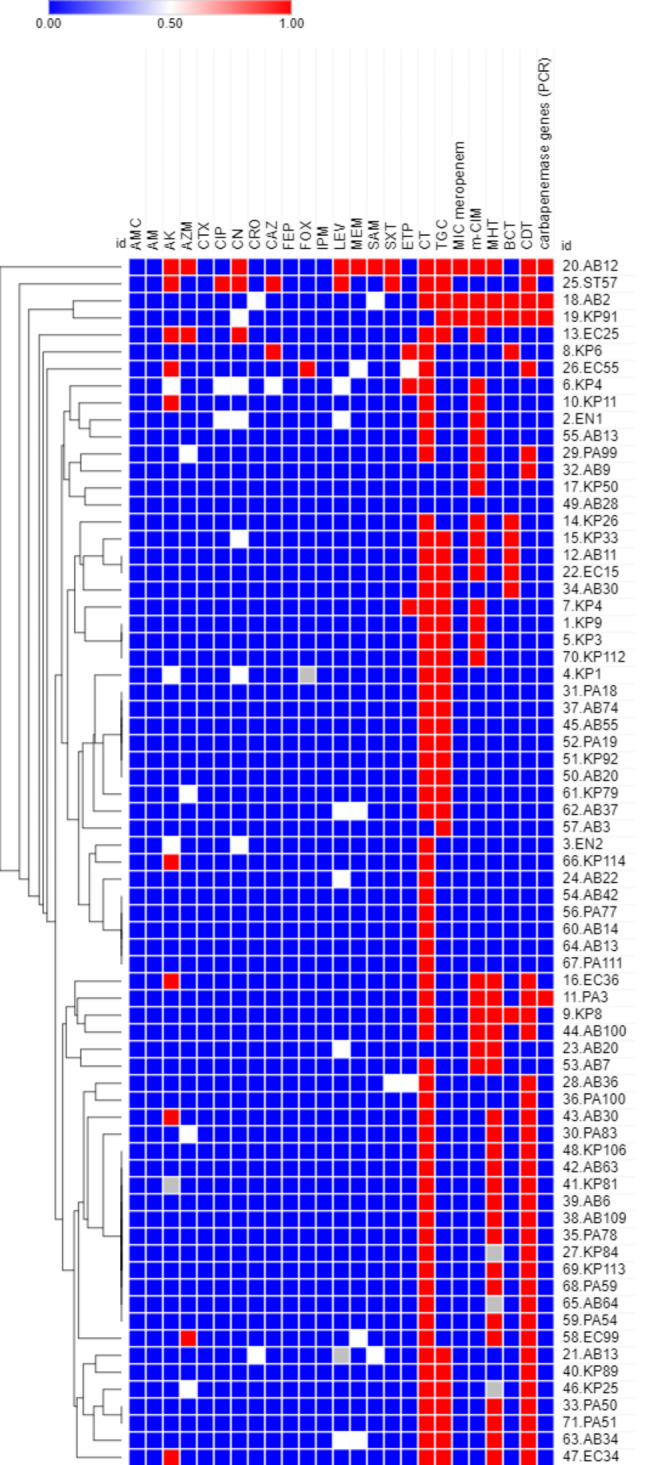
The heat-map clonal analysis of the among the recovered carbapenem-resistance Gram-negative bacilli (CR-GNB) phenotypes. AMC (amoxicillin/clavulanic acid), AM (ampicillin), AK (amikacin), AZM (aztreonam), CTX (cefotaxime), CIP (ciprofloxacin), CRO (ceftriaxone), CAZ (ceftazidime), FEP (cefepime), FOX (cefoxitin), (CT) colistin, ETP (ertapenem), CN (gentamicin), IPM (imipenem), LEV (levofloxacin), (MEM) meropenem), (SAM) ampicillin/ sulbactam, SXT (trimethoprim / sulfamethoxazole, TGC (tigecycline). MHT (modified Hodge test), mCIM (modified carbapenem inactivation method), CDT, (combined disk test by EDTA), BCT (blue-carba test)

Heat-map showing different antimicrobial resistance patterns and carbapenemase enzymes (BCT, CDT, mCIM, MHT, PCR) among 71 CR-GNB (Fig. 3). The heat map analysis of the 71 CR-GNB isolates showed that they were not clonal (Fig. 3).

**Table 2:** Statistical association between carbapenemase enzyme and phenotypic tests with their respective *p* values

Pearson Chi-Square (two-tailed *P* value)						
Carbapenamase enzyme	Phenotypic Tests	*Enterobacterales*	*A. baumannii*	*P. aeruginosa*	Non-glucose fermenting GNB	Total tested isolates
*bla*_KPC_	MHT	P = 0.334	P = 0.126	P = 0.428	P = 0.116	P = 0.096
	mCIM	P = 0.525	P = 0.573	P = 0.715	P = 0.475	P = 0.500
	BCT	P = 0.084	P = 0.693	P = 0.188	P = 0.261	P = 0.088
	CDT	P = 0.192	P = 0.136	P = 0.071	P = 0.021*	P = 0.020*
*bla*_NDM_	MHT	P = 0.053	P = 0.145	NA	P = 0.121	P = 0.009*
	mCIM	P = 0.159	P = 0.881	NA	P = 0.707	P = 0.684
	BCT	P = 0.631	P = 0.193	NA	P = 0.072	P = 0.464
	CDT	P = 0.017*	P = 0.100	NA	P = 0.052	P = 0.001*
*bla*_VIM_	MHT	P = 0.039*	P = 0.005*	P = 0.014*	P = 0.006*	P = 0.000*
	mCIM	P = 0.056	P = 0.738	P = 0.273	P = 0.526	P = 0.350
	BCT	P = 0.732	P = 0.136	NA	P = 0.159	P = 0.943
	CDT	P = 0.033*	P = 0.001*	P = 0.030*	P = 0.004*	P = 0.000*
*bla*_GIM_	MHT	P = 0.519	P = 0.727	P = 0.260	P = 0.738	P = 0.615
	mCIM	P = 0.309	P = 0.512	P = 0.657	P = 0.707	P = 0.523
	BCT	P = 0.041*	P = 0.603	NA	P = 0.613	P = 0.446
	CDT	P = 0.406	P = 0.188	P = 0.118	P = 0.052	P = 0.048*
*bla*_OXA−48_	MHT	P = 0.717	P = 0.003*	P = 0.428	P = 0.061	P = 0.115
	mCIM	P = 0.269	P = 0.472	P = 0.715	P = 0.319	P = 0.099
	BCT	P = 0.140	P = 0.495	NA	P = 0.63	P = 0.305
	CDT	P = 0.313	P = 0.816	P = 0.071	P = 0.277	P = 0.287

* Statistically significant, MHT, modified Hodge test, mCIM, modified carbapenem inactivation method, CDT, combined disk test by EDTA ; BCT, blue-carba test. Non glucose fermenting bacilli included *A. baumannii, P. aeruginosa* and *Stenotrophomonas maltophilia* isolates. NA: not applicable

The statistical association between carbapenemase enzyme and phenotypic tests with their respective *p* values was shown in Table 2. The results had indicated that CPO having *bla*_KPC_ or *bla*_GIM_ genotypes had shown a statistical significance with CDT by EDTA at *p* value 0.02 and 0.04, respectively. The CPO having *bla*_NDM_ and *bla*_VIM_ had also shown statistically significant relation with CDT by EDTA and MHT. Isolates producing *bla*_OXA−48_ did not show statistical correlation with any of the tested phenotypic tests. Strength of Spearman’s correlation coefficient among all possible genotypes and phenotypes combinations was shown in Table 3. The results revealed a significant Spearman’s correlation coefficient (0.515–0.774) between *bla*_NDM_ and/or *bla*_VIM_ genotypes with isolates giving positive results with MHT and/or CDT by EDTA (supplementary file, Table S2-S8).

**Table 3:** Spearman correlation coefficient among all possible genotypes and phenotypes combinations

Sample 1	Sample 2	Correlation	95% CI for ρ	*P-*Value
*bla*_VIM_ + *bla*_GIM_ + *bla*_NDM_	MHT + BCT + EDTA	0.774	[64.0- 86.2]	0
*bla*_VIM_ + *bla*_NDM_	MHT + EDTA	0.763	[62.5–85.5]	0
*bla*_VIM_ + *bla*_GIM_ + *bla*_NDM_	EDTA + mCIM + MHT	0.736	[58.7–83.6]	0
*bla*_VIM_ + *bla*_NDM_	EDTA	0.724	[0.57.1–82.8]	0
*bla*_VIM_ + *bla*_NDM_	MHT + BCT + EDTA	0.718	[0.56.3–82.4]	0
*bla*_VIM_ + *bla*_GIM_ + *bla*_NDM_	MHT	0.711	[54.8–0.82.1]	0
*bla*_VIM_ + *bla*_NDM_	EDTA + mCIM + MHT	0.683	[51.6–0.80.0]	0
*bla*_VIM_ + *bla*_NDM_	MHT	0.679	[50.7–80.0]	0
*bla*_VIM_ + *bla*_GIM_ + *bla*_NDM_	BCT + EDTA	0.678	[50.9–79.7]	0
*bla*_VIM_ + *bla*_GIM_ + *bla*_NDM_	MHT + BCT + EDTA + mCIM	0.653	[47.7–77.9]	0
*bla*_VIM_ + *bla*_GIM_ + *bla*_NDM_ + *bla*_OXA−48_	MHT	0.653	[47.3–78.1]	0
*bla*_VIM_ + *bla*_NDM_	MHT + BCT + EDTA + mCIM	0.623	[43.8–75.7]	0
*bla*_VIM_ + *bla*_GIM_ + *bla*_NDM_ + *bla*_OXA−48_	MHT + EDTA	0.617	[43.1–75.3]	0
*bla*_VIM_ + *bla*_GIM_ + *bla*_NDM_ + *bla*_OXA−48_	EDTA + mCIM + MHT	0.61	[42.1–74.7]	0
*bla*_VIM_ + *bla*_NDM_	Blue Carba + EDTA	0.607	[41.8–74.6]	0
*bla*_VIM_ + *bla*_NDM_ + *bla*_OXA−48_	MHT	0.6	[40.4–74.3]	0
*bla*_VIM_ + *bla*_GIM_ + *bla*_NDM_ + *bla*_OXA−48_	MHT + BCT + EDTA	0.593	[40.1–73.5]	0
*bla*_VIM_ + *bla*_GIM_	MHT + EDTA	0.581	[38.6–72.6]	0
*bla*_VIM_ + *bla*_GIM_ + *bla*_NDM_	mCIM + EDTA	0.581	[38.6–72.6]	0
*bla*_VIM_ + *bla*_GIM_	EDTA	0.577	[38.1–72.3]	0
*bla*_VIM_ + *bla*_NDM_	MHT + BCT	0.559	[35.9–71.0]	0
*bla*_VIM_ + *bla*_GIM_ + *bla*_NDM_	MHT + BCT	0.558	[35.8–70.9]	0
*bla*_VIM_ + *bla*_GIM_ + *bla*_NDM_ + *bla*_OXA−48_	MHT + BCT + EDTA + mCIM	0.558	[35.8–70.9]	0
*bla*_VIM_ + *bla*_GIM_	MHT + BCT + EDTA	0.548	34.6–70.2]	0
*bla*_VIM_ + *bla*_GIM_ + *bla*_NDM_ + *bla*_OXA−48_	MHT + BCT	0.544	[34.1–69.8]	0
*bla*_VIM_ + *bla*_NDM_ + *bla*_OXA−48_	EDTA + mCIM + MHT	0.541	[33.8–69.7]	0
*bla*_VIM_ + *bla*_NDM_ + *bla*_OXA−48_	MHT + EDTA	0.533	[32.8–69.0]	0
*bla*_VIM_ + *bla*_NDM_ + *bla*_OXA−48_	MHT + BCT + EDTA	0.524	[31.7–68.3]	0
*bla*_VIM_ + *bla*_NDM_	mCIM + EDTA	0.523	[31.6–68.2]	0
*bla*_VIM_ + *bla*_NDM_ + *bla*_OXA−48_	MHT + BCT	0.518	[31.0- 67.9)	0
*bla*_VIM_	MHT + EDTA	0.517	[30.7–67.9]	0
*bla*_VIM_ + *bla*_GIM_ + *bla*_NDM_ + *bla*_OXA−48_	mCIM + MHT	0.515	[30.6–67.6]	0
*bla*_VIM_ + *bla*_GIM_ + *bla*_NDM_ + *bla*_OXA−48_	EDTA	0.515	[30.7–67.7]	0
*bla*_VIM_ + *bla*_NDM_ + *bla*_OXA−48_	MHT + BCT + EDTA + mCIM	0.509	[29.9–67.2]	0
*bla*_VIM_	MHT + BCT + EDTA	0.502	[29.0- 66.8]	0

*Bla*_KPC_, *gene coded for Klebsiella pneumoniae* carbapenemases (KPC); *bla*_NDM_, a gene coded for New Delhi metallo-β-lactamase (NDM); imipenem-resistant *Pseudomonas*-type carbapenemases (IMP); *bla*_VIM_, a gene coded for Verona integron-encoded metallo-β-lactamase (VIM); *bla*_GIM_, a gene coded for German imipenemase and *bla*_OXA−48,_ oxacillinase (OXA-48-like) types. MHT, modified Hodge test, mCIM, modified carbapenem inactivation method, CDT, combined disk test by EDTA; BCT, blue-carba test

## Discussion

Given their alarming prevalence across the globe including Egypt, infections caused by CR-GNB especially CPO poses an ongoing public health threat that is associated with higher mortality rates, longer hospitalization and increased healthcare costs [26]. Of note, World Health Organization (WHO) had listed CR-NF namely *A. baumannii* complex and *P. aeruginosa* along with CRE as critical pathogens with the most tenacious resistant problem for which innovative new treatments are urgently required [27]. Therefore, this study was conducted to determine the significant association of the molecular and phenotypic tests to determine the most reliable phenotypic test to guide the physician in selecting the appropriate empirical antibiotic therapy, particularly in life-threatening infections such as meningitis and pneumonia or bloodstream infections caused by this nightmare pathogens.

In this study, we aimed to detect the prevalence of CPO across 71 CR-GNB, after checking their resistance profiles by disk diffusion method and determining potential CPO by broth microdilution assay against meropenem antibiotic. Our results revealed that the previously mentioned CR-NF along with *K. pneumoniae* were the most frequently isolated, reflecting their bioburden and ensuring their importance as life threating human pathogens. The antibiogram analysis of CR-GNB clinical isolates revealed high resistance pattern to β-lactam group, aminoglycosides, quinolones and sulfonamides/diaminopyrimidines while, colistin and tigecycline had shown improved susceptibility pattern. Additionally, 98.6% of tested CR-GNB expressed MDR phenotypes, which was in accordance with other recently published national studies that underscore the considerable resistant of CPO [28, 29]. Our analysis of the meropenem MIC profile among distinct bacterial genera revealed that majority of CRE (29/30, 96.66%) and CR-NF (39/41, 95.12%) isolates displayed MIC values of ≥ 2–4 µg/ml and ≥ 4–8 µg/ml, respectively and were considered as potential CPO as recommended by CLSI. Despite of meropenem enhanced performance as an initial screening test, still carbapenemase enzymes as *bla*_OXA−48_ and variants of *bla*_KPC_ with weak activity against carbapenems can be underestimated [30–32].

Of concern, the dissemination of these perturbing enzymes including OXA−48, KPC and class B metallo β-lactamases within our hospital settings had called for prompt detection of CPO to allow implementing effective infection control measures and prescribing of appropriate antimicrobial regimen [33, 34]. In context of this, a series of phenotypic tests including MHT, mCIM, BCT and CDT was proposed to evaluate their performance, in comparison to gold standard PCR. Our study revealed that MHT had shown relatively reduced sensitivity and low NPV in detecting CPE (74%, 12.50%) and CP-NF (62.16%, 17.64%), respectively. Such finding could be attributed to failure of MHT to detect 21 CPO isolates (7 CPE and 14 CP-NFB) that carry *bla*_KPC_ either alone or in combination with *bla*_OXA−48_ [1, 14]. However, Yan et al. had improved the efficiency of the MHT in detecting KPC-producing *K. pneumoniae* mucoid colonies by using EDTA that primarily lyses bacterial cells to release β-lactamases [35].

To overcome pitfalls of MHT in terms of its subjective interpretation and validity, CLSI had endorsed a more reliable growth based carbapenemase detection test, mCIM. In comparison to MHT, mCIM had shown improved sensitivity in the detection of total CPO (68.65%) and CP-NF (81.75%). Our findings were in tune with Kuchibiro et al., who reported on improved sensitivity of mCIM in detecting CP-*A. baumannii* and CP-*Pseudomonas* spp. at 76.5% and 90%, respectively [36]. In this study, the decreased sensitivity and NPV of mCIM could be related to high number of false negative results (14 CPE and 7 CP-NF), whereas 16 of these strains (10 CPE and 6 CP-NF) produced *bla*_KPC_, accounting for 76.19%. Of note, such isolates produced either *bla*_OXA−48_ or class B carbapenemase along with *bla*_KPC_. This data suggest that further studies could be required to estimate effect of longer incubation period between tested isolate and meropenem disk (> 4 h) to ensure detection of carbapenemases with either low level of expression, weak hydrolytic activities, or class B carbapenemase that requires divalent cations for their action.

Metallo-β-lactamases of subclass B1 as *bla*_VIM_ and *bla*_NDM_ are resistance determinant of increased clinical relevance within our hospital settings. The CDT an inhibitor-based approach that depends on suppressing the enzymatic activity by a chelating agent namely EDTA was tested, prior to PCR. In comparison to MHT and mCIM, CDT by EDTA had shown a reduced sensitivity rate for detection of CPE, CP-NF and CPO at 62.07%, 50% and 55.22%, respectively. Out of 30 false negative results, 27 (90%) carry *bla*_KPC_ alone or in combination with *bla*_OXA−48_, that were not inhibited by EDTA. Of particular concern that the other 3 false negative results belong to CP-NF (*A. baumannii*, *P. aeruginosa*, *Stenotrophomonas maltophilia*) that co-produce *bla*_KPC_ + *bla*_VIM_ + *bla*_OXA−48_. Failure to detect *bla*_VIM_ in such isolates could be attributed to inability of EDTA to reach metallo β-lactamase active site that is characterized by being a shallow groove with few contact points [37] or it could be related to inadequate expression of *bla*_VIM_.

Lately, a variety of colorimetric methods that explore hydrolytic activity of carbapenemase enzymes within a short turnaround time as Carba-NP and its variant BCT had been proposed. In comparison to PCR, the colorimetric assays always-broader detection of carbapenemase activity and it is a reliable method for rapid detection of CPO directly from solid media or blood cultures [38].

Among the other three evaluated phenotypic tests in this study, BCT had presented the highest sensitivity for detection of CPE, CP-NF, and CPO at 82.75%, 94.74% and 89.55%, respectively. Our results were nearly in agreement with other studies that reported on higher sensitivity of BCT for detection of CPE ranging from 95.3 to 98% [39, 40]. However, a relatively lower specificity (66.66%) and PPV (97.29%) were recorded for BCT among CR- NG in our study. This could be attributed to false positive result exhibited by an *A. baumannii* isolate that carry either *bla*_OXA−48_ variants or rare carbapenemase not detected by PCR. On the contrary, no false positive results were recorded with the other three phenotypic tests, rendering perfect specificity and PPV.

Another important aim of our study was to evaluate the correlation between CR genes and different phenotypic tests. Our results recorded a statistically significant difference between CPO isolates having class B carbapenemases and/or *bla*_KPC_ with CDT by EDTA. Although previous studies had reported on effectiveness of EDTA to inhibit metallo β-lactamase enzymes [41, 42], still there is paucity of data on its capability to interact with *bla*_KPC_. However, we should put in consideration that EDTA itself effect membrane permeabilization particularly in non-fermenting bacilli and restore activity of carbapenem leading to false interpretation of CDT [43, 44]. Additionally, CPO having *bla*_VIM_ or *bla*_NDM_ had shown statistically significant difference with MHT. This results were comparable to Sultan et al., who reported on effectiveness of MHT to determine *bla*_NDM_ in clinical laboratories of Pakistan [45]. Finally, *Enterobacterales* isolates producing *bla*_GIM_ had shown significant statistical association with BCT. In summary, each phenotypic test has its own pros and cons. However, limitation of study includes the inability to evaluate performance of Carba NP and presence of isolates harboring more than one type of carbapenemase enzymes that may impede performance of CDT with EDTA. Another limitation is evaluating performance of MHT that is not currently recommended by CLSI.

## Conclusion

The performance of phenotypic tests (sensitivity/specificity/PPV/NPV) varied depending on bacterial genera, genotype, and coexistence of more than one type of carbapenemase. Statistical analysis showed significant association (*p <* 0.05) between *bla*_NDM_/*bla*_VIM_ genotypes with MHT/CDT; *bla*_KPC_/*bla*_GIM_ genotypes with CDT and *bla*_GIM_ genotype with BCT. BCT possess a relatively high performance for the efficient and rapid detection of CPO. The overall sensitivity/specificity values for detection of CPO were 65.62%/100% for MHT, 68.65%/100% for mCIM, 55.22%/100% for CDT and 89.55%/75% for BCT. Based on its respective diagnostic efficiency and rapid turnaround time, BCT is more likely to be recommended in our resource limited settings when PCR is not available.

## Electronic supplementary material

Below is the link to the electronic supplementary material.

Supplementary Material 1

## Data Availability

All data generated or analyzed during this study are included in this published article in the main manuscript.

## List of abbreviations

*Bla*_KPC_: the gene coded for *Klebsiella pneumoniae* carbapenemases (KPC).
*bla*_NDM_: the gene coded for New Delhi metallo-β-lactamase (NDM).
*Bla*_IMP_: the gene coded for the imipenem-resistant *Pseudomonas*-type carbapenemases (IMP).
*bla*_VIM_: a gene coded for Verona integron-encoded metallo-β-lactamase (VIM).
*bla*_GIM_: the gene coded for German imipenemase
*bla*_OXA−48_: the gene coded oxacillinase (OXA-48-like) types
BCT: blue-carba test
CLSI: clinical and laboratory standard institute
CDT: combined disk test by EDTA
CPGN: Carbapenemase-producing Gram-negative (CPGN)
CPO: Carbapenemase-producing organisms
EDTA: ethylene diamine tetra acetic acid
GNB: Gram-negative bacilli
MDR: mlti-drug resistant
MHT: modified Hodge test
mCIM: modified carbapenem inactivation method
FP: False positive
FN: False negative
TP: true positive
TN: true negative
PPV: Positive predictive value
NPV: Negative predictive value
PCR: polymerase chain reaction

## References

[1] Zhang Z, Wang D, Li Y, Liu Y, Qin X (2022). Comparison of the Performance of Phenotypic Methods for the Detection of Carbapenem-Resistant Enterobacteriaceae (CRE) in Clinical Practice. Front Cell Infect Microbiol.

[2] Armstrong T, Fenn SJ, Hardie KR. JMM Profile: Carbapenems: a broad-spectrum antibiotic. J Med Microbiol. 2021;70(12):001462. 10.1099/jmm.0.001462. PMID: 34889726. 10.1099/jmm.0.001462 PMC8744278 34889726

[3] Chia PY, Sengupta S, Kukreja A, Ponnampalavanar SL, Ng S, Marimuthu OT. K. The role of hospital environment in transmissions of multidrug-resistant gram-negative organisms. Antimicrob Resist Infect Control. 2020 Feb 11;9(1):29. 10.1186/s13756-020-0685-1. 10.1186/s13756-020-0685-1 PMC7014667 32046775

[4] Sheu C-C, Chang Y-T, Lin S-Y, Chen Y-H, Hsueh P-R (2019). Infections Caused by Carbapenem-Resistant Enterobacteriaceae: An Update on Therapeutic Options. Front Microbiol.

[5] Suay-García B, Pérez-Gracia MT (2019). Present and Future of Carbapenem-resistant Enterobacteriaceae (CRE) Infections. Antibiot (Basel).

[6] Nordmann P, Poirel L (2014). The difficult-to-control spread of carbapenemase producers among Enterobacteriaceae worldwide. J Clin Microbiol Infect.

[7] Moawad AA, Hotzel H, Hafez HM, Ramadan H, Tomaso H, Braun SD, Ehricht R, Diezel C, Gary D, Engelmann I, Zakaria IM, Reda RM, Eid S, Shahien MA, Neubauer H, Monecke S (2022). Occurrence, Phenotypic and Molecular Characteristics of Extended-Spectrum Beta-Lactamase-Producing *Escherichia coli* in Healthy Turkeys in Northern Egypt. Antibiot (Basel).

[8] Hadjadj L, Syed MA, Abbasi SA, Rolain JM, Jamil B (2021). Diversity of Carbapenem Resistance Mechanisms in Clinical Gram-Negative Bacteria in Pakistan. Microb Drug Resist.

[9] Wilson GM, Suda KJ, Fitzpatrick MA, Bartle B, Pfeiffer CD, Jones M, Rubin MA, Perencevich E, Evans M, Evans CT (2021). QUERI CARRIAGE Program. Risk Factors Associated With Carbapenemase-Producing Carbapenem-Resistant Enterobacteriaceae Positive Cultures in a Cohort of US Veterans. Clin Infect Dis.

[10] Ramsamy Y, Mlisana KP, Amoako DG, Abia ALK, Ismail A, Allam M, Mbanga J, Singh R, Essack SY (2022). Mobile genetic elements-mediated *Enterobacterales*-associated carbapenemase antibiotic resistance genes propagation between the environment and humans: A One Health South African study. Sci Total Environ.

[11] Kopotsa K, Osei Sekyere J, Mbelle NM (2019). Plasmid evolution in carbapenemase-producing Enterobacteriaceae: a review. Ann N Y Acad Sci.

[12] Otsuka Y. Potent Antibiotics Active against Multidrug-Resistant Gram-Negative Bacteria. Chem Pharm Bull (Tokyo). 2020;68(3):182–190. 10.1248/cpb.c19-00842. PMID: 32115524. 10.1248/cpb.c19-00842 32115524

[13] Ma CW, Ng KK, Yam BH, Ho PL, Kao RY, Yang D. Rapid Broad Spectrum Detection of Carbapenemases with a Dual Fluorogenic-Colorimetric Probe. J Am Chem Soc. 2021 May 12;143(18):6886–6894. 10.1021/jacs.1c00462. 10.1021/jacs.1c00462 33909441

[14] Workneh M, Yee R, Simner PJ (2019). Phenotypic Methods for Detection of Carbapenemase Production in Carbapenem-Resistant Organisms: What Method Should Your Laboratory Choose?. Clin Microbiol Newsletter.

[15] Vamsi SK, Moorthy RS, Hemiliamma MN, Chandra Reddy RB, Chanderakant DJ, Sirikonda S (2022). Phenotypic and genotypic detection of carbapenemase production among gram negative bacteria isolated from hospital acquired infections. Saudi Med J.

[16] He J, Du X, Zeng X, Moran RA, van Schaik W, Zou Q, Yu Y, Zhang J, Hua X (2022). Phenotypic and Genotypic Characterization of a Hypervirulent Carbapenem-Resistant Klebsiella pneumoniae ST17-KL38 Clinical Isolate Harboring the Carbapenemase IMP-4. Microbiol Spectr.

[17] CLSI: Performance Standards for Antimicrobial Susceptibility Testing, 31th Ed. edn. Wayne, PA.: Clinical and Laboratory Standards Institute.; 2021. https://clsi.org/standards/products/microbiology/documents/m100/. Accessed 20 April 2022

[18] Bergey D, Holt J: Bergey’s manual of determinative bacteriology., vol. 527: Williams & Wilkins: Baltimore, MD, USA, 1994. https://www.worldcat.org/title/bergeys-manual-of-determinative-bacteriology/oclc/28183643. Accessed 20 April 2022

[19] Magiorakos AP, Srinivasan A, Carey RB, Carmeli Y, Falagas ME, Giske CG, Harbarth S, Hindler JF, Kahlmeter G, Olsson-Liljequist B, Paterson DL, Rice LB, Stelling J, Struelens MJ, Vatopoulos A, Weber JT, Monnet DL (2012). Multidrug-resistant, extensively drug-resistant and pandrug-resistant bacteria: an international expert proposal for interim standard definitions for acquired resistance. Clin Microbiol Infect.

[20] CLSI: Performance Standards for Antimicrobial Susceptibility Testing, 27th Ed. edn. Wayne, PA.: Clinical and Laboratory Standards Institute; 2017. https://clsi.org/media/1469/m100s27_sample.pdf

[21] Pires P, Novais J, Peixe A (2013). Blue-carba, an easy biochemical test for detection of diverse carbapenemase producers directly from bacterial cultures. J Clin Microbiol.

[22] Pasteran F, Veliz O, Ceriana P, Lucero C, Rapoport M, Albornoz E, Gomez S, Corso A (2015). Evaluation of the Blue-Carba test for rapid detection of carbapenemases in gram-negative bacilli. J Clin Microbiol.

[23] Nastro M, Ayora M, García S, Vay C, Famiglietti Á, Rodriguez CH (2017). Rapid Blue-Carba test: reduction in the detection time of carbapenemases performed from a 4-hour bacterial lawn. J Chemother.

[24] Pournaras S, Zarkotou O, Poulou A, Kristo I, Vrioni G, Themeli-Digalaki K, Tsakris A (2013). A combined disk test for direct differentiation of carbapenemase-producing enterobacteriaceae in surveillance rectal swabs. J Clin Microbiol.

[25] Kamel NA, Elsayed KM, Awad MF, Aboshanab KM, Borhamy E. MI. Multimodal Interventions to Prevent and Control Carbapenem-Resistant Enterobacteriaceae and Extended-Spectrum beta-Lactamase Producer-Associated Infections at a Tertiary Care Hospital in Egypt. Antibiot (Basel) 2021;10. 10.3390/antibiotics10050509. 10.3390/antibiotics10050509 PMC8146387 33946253

[26] Sleiman A, Fayad AGA, Banna H, Matar GM (2021). Prevalence and molecular epidemiology of carbapenem-resistant Gram-negative bacilli and their resistance determinants in the Eastern Mediterranean Region over the last decade. J Glob Antimicrob Resist.

[27] WHO: Global Priority List of Antibiotic-Resistant Bacteria to Guide Research, Discovery, and Development of New Antibiotics. 2017. https://www.who.int/news/item/27-02-2017-who-publishes-list-of-bacteria-for-which-new-antibiotics-are-urgently-needed. Accessed 22 April 2022

[28] Elshamy AA, Saleh SE, Alshahrani MY, Aboshanab KM, Aboulwafa MM, Hassouna NA. OXA-48 Carbapenemase-Encoding Transferable Plasmids of *Klebsiella pneumoniae* Recovered from Egyptian Patients Suffering from Complicated Urinary Tract Infections. Biology 2021; 10(9). 10.3390/biology10090889 PMC8469419 34571766

[29] Mabrouk SS, abdellatif g, El-Ansary MR, Aboshanab KMA, Ragab YMJI, Resistance D. Carbapenemase Producers Among Extensive Drug-Resistant Gram-Negative Pathogens Recovered from Febrile Neutrophilic Patients in Egypt. 2020; 13:3113–3124. 10.2147/IDR.S269971 PMC7495499 32982326

[30] Tamma T, Wang PD, Lewis R, Opene S, Simner BNA (2017). Is There a Carbapenem MIC Cutoff Value That Distinguishes Carbapenemase-Producing and Non-Carbapenemase-Producing Carbapenem Non-Susceptible *Pseudomonas* and *Acinetobacter* Isolates?. Infect Control Hosp Epidemiol.

[31] Kidd JM, Livermore DM, Nicolau DP (2020). The difficulties of identifying and treating *Enterobacterales* with OXA-48-like carbapenemases. Clin Microbiol Infect.

[32] Cui X, Zhang H, Du H. Carbapenemases in Enterobacteriaceae: Detection and Antimicrobial Therapy. Front Microbiol. 2019 Aug 20;10:1823. 10.3389/fmicb.2019.01823. 10.3389/fmicb.2019.01823 PMC6710837 31481937

[33] El-Kholy AA, Girgis SA, Shetta MAF, Abdel-Hamid DH, Elmanakhly AR (2020). Molecular characterization of multidrug-resistant Gram-negative pathogens in three tertiary hospitals in Cairo, Egypt. Eur J Clin Microbiol Infect Dis.

[34] Makharita RR, El-Kholy I, Hetta HF, Abdelaziz MH, Hagagy FI, Ahmed AA, Algammal AM (2020). Antibiogram and Genetic Characterization of Carbapenem-Resistant Gram-Negative Pathogens Incriminated in Healthcare-Associated Infections. Infect Drug Resist.

[35] Yan Y, Yang H, Pan L, Sun K, Fan H, Lu Y, Shi Y (2014). Improving the Efficiency of the Modified Hodge Test in KPC-Producing Klebsiella pneumoniae Isolates by Incorporating an EDTA Disk. Curr Microbiol.

[36] Kuchibiro T, Komatsu M, Yamasaki K, Nakamura T, Nishio H, Kimura K, Niki M, Kida K, Ohama M, Fukuda N (2021). Comparison of the performance of three carbapenem inactivation methods for the detection of carbapenemase-producing gram-negative bacilli. J infect Chemother.

[37] Mojica MF, Rossi MA, Vila AJ, Bonomo RA (2022). The urgent need for metallo-β-lactamase inhibitors: an unattended global threat. Lancet Infect Dis.

[38] Meier M, Hamprecht A. Systematic Comparison of Four Methods for Detection of Carbapenemase-Producing *Enterobacterales* Directly from Blood Cultures. *J Clin Microbiol.* 2019 23;57(11):e00709-19. 10.1128/JCM.00709-19. 10.1128/JCM.00709-19 PMC6813004 31413083

[39] Pancotto LR, Nodari CS, Rozales FP, Soldi T, Siqueira CG, Freitas AL, Barth AL (2018). Performance of rapid tests for carbapenemase detection among Brazilian Enterobacteriaceae isolates. Braz J Microbiol.

[40] Tanriverdi Cayci Y, Biyik I, Korkmaz F, Birinci A (2021). Investigation of NDM, VIM, KPC and OXA-48 genes, blue-carba and CIM in carbapenem resistant *Enterobacterales* isolates. J Infect Dev Ctries.

[41] Palacios AR, Rossi MA, Mahler GS, Vila AJ (2020). Metallo-β-Lactamase Inhibitors Inspired on Snapshots from the Catalytic Mechanism. Biomolecules.

[42] Naim H, Rizvi M, Gupta R, Azam M, Taneja N, Shukla I, Khan HM (2018). Drug Resistance and Molecular Epidemiology of Carbapenem Resistant Gram-negative Bacilli Isolates. J Glob Infect Dis.

[43] Bedenić B, Ladavac R, Vranić-Ladavac M, Barišić N, Karčić N, Sreter KB, Mihaljević S, Bielen L, Car H, Beader N (2019). False positive phenotypic detection of metallo-beta-lactamases in *Acinetobacter baumannii*. Acta clin Croat.

[44] Josa MD, Leal R, Rojas J, Torres MI, Cortés-Muñoz F, Esparza G, Reyes LF. Comparative Evaluation of Phenotypic Synergy Tests versus RESIST-4 O.K.N.V. and NG Test Carba 5 Lateral Flow Immunoassays for the Detection and Differentiation of Carbapenemases in *Enterobacterales* and *Pseudomonas aeruginosa.* Microbiol Spectr. 2022 Feb 23;10(1):e0108021. 10.1128/spectrum.01080-21. 10.1128/spectrum.01080-21 PMC8809327 35107384

[45] Sultan BA, Khan E, Hussain F, Nasir A, Irfan SJJPMA (2013). Effectiveness of modified Hodge test to detect NDM-1 carbapenemases: an experience from Pakistan. J Pak Med Assoc.

