# Supplementary material for: Insights on the performance of phenotypic tests versus genotypic tests for the detection of carbapenemase-producing Gram-negative bacilli in resource-limited settings
Source: BMC Microbiol. 2022 Oct 14;22:248. doi: 10.1186/s12866-022-02660-5 (PMC9563167; doi:10.1186/s12866-022-02660-5)
Supplement: Supplementary file 1 — Supplementary Material 1 [file 12866_2022_2660_MOESM1_ESM.docx]

**Table S1**: Primers, amplicon size and annealing temperatures (Ta) of the tested carbapenemase genes.

| Ambler classification | Carbapenemase genes | Nucleotide Sequence (5’-3’) primers | Amplicon size (bp) | Ta (ºC) |
| --- | --- | --- | --- | --- |
| Class A | *bla*_KPC_ | P_F_ TGTCACTGTATCGCCGTC  P_R_ CTCAGTGCTCTACAGAAAACC | 1011 | 50 |
| Class B | *bla*_GIM_ | P_F_ TCGACACACCTTGGTCTGAA  P_R_ AACTTCCAACTTTGCCATGC | 477 | 52 |
|  | *bla*_VIM_ | P_F_ TCTACATGACCGCGTCTGTC  P_R_- TGTGCTTTGACAACGTTCGC | 748 | 50 |
|  | *bla*_NDM_ | P_F_ GGTTTGGCGATCTGGTTTTC  P_R_ CGGAATGGCTCATCACGATC | 621 | 50 |
| Class D | *bla*_OXA-48_ | P_F_ GCGTGGTTAAGGATGAACAC  P_R_ CATCAAGTTCAACCCAACCG | 438 | 50 |

**Table S2:** contingency table for chi-square calculations

|  |  |  | *bla*_KPC_ | | *bla*_NDM_ | | *bla*_VIM_ | | *Bla*_GIM_ | | *bla*_OXA-48_ | |
| --- | --- | --- | --- | --- | --- | --- | --- | --- | --- | --- | --- | --- |
| Phenotypic test | Tested microorganism |  | -Ve | + Ve | -  Ve | + Ve | -  Ve | + Ve | -  Ve | + Ve | -  Ve | + Ve |
| m-CIM | *Enterobacterales* | -Ve | 4 | 10 | 13 | 2 | 7 | 7 | 14 | 1 | 10 | 5 |
|  |  | +Ve | 3 | 13 | 9 | 5 | 11 | 2 | 15 | 0 | 7 | 8 |
|  | Non- glucose fermenting | -Ve | 5 | 6 | 9 | 1 | 7 | 2 | 9 | 1 | 6 | 4 |
|  |  | +Ve | 10 | 13 | 29 | 2 | 20 | 10 | 29 | 2 | 13 | 18 |
|  | Total | -Ve | 9 | 16 | 22 | 3 | 14 | 9 | 23 | 2 | 16 | 9 |
|  |  | +Ve | 13 | 33 | 38 | 7 | 31 | 12 | 44 | 2 | 20 | 26 |
|  |  |  |  |  |  |  |  |  |  |  |  |  |
| MHT | *Enterobacterales* | -Ve | 1 | 7 | 8 | 0 | 7 | 0 | 8 | 0 | 5 | 3 |
|  |  | +Ve | 6 | 14 | 13 | 7 | 11 | 8 | 19 | 1 | 11 | 9 |
|  | Non- glucose fermenting | -Ve | 4 | 13 | 17 | 0 | 16 | 1 | 16 | 1 | 11 | 6 |
|  |  | +Ve | 11 | 12 | 20 | 3 | 9 | 13 | 21 | 2 | 8 | 15 |
|  | Total | -Ve | 5 | 20 | 25 | 0 | 23 | 1 | 24 | 1 | 16 | 9 |
|  |  | +Ve | 17 | 26 | 33 | 10 | 20 | 21 | 40 | 3 | 19 | 24 |
|  |  |  |  |  |  |  |  |  |  |  |  |  |
| BCT | *Enterobacterales* | -Ve | 3 | 3 | 5 | 1 | 6 | 2 | 5 | 1 | 5 | 1 |
|  |  | +Ve | 4 | 20 | 17 | 6 | 13 | 6 | 24 | 0 | 12 | 12 |
|  | Non- glucose fermenting | -Ve | 2 | 1 | 2 | 1 | 0 | 1 | 3 | 0 | 1 | 2 |
|  |  | +Ve | 13 | 25 | 36 | 2 | 25 | 12 | 35 | 3 | 18 | 20 |
|  | Total | -Ve | 5 | 4 | 7 | 2 | 6 | 3 | 8 | 1 | 6 | 3 |
|  |  | +Ve | 17 | 45 | 53 | 8 | 38 | 18 | 59 | 3 | 30 | 32 |
|  |  |  |  |  |  |  |  |  |  |  |  |  |
| CDT by EDTA | *Enterobacterales* | -Ve | 1 | 11 | 11 | 0 | 10 | 0 | 12 | 0 | 7 | 6 |
|  |  | +Ve | 5 | 13 | 11 | 7 | 11 | 6 | 17 | 1 | 10 | 7 |
|  | Non- glucose fermenting | -Ve | 5 | 17 | 22 | 0 | 18 | 3 | 22 | 0 | 8 | 13 |
|  |  | +Ve | 11 | 8 | 16 | 3 | 5 | 12 | 16 | 3 | 11 | 9 |
|  | Total | -Ve | 6 | 28 | 33 | 0 | 28 | 3 | 34 | 0 | 15 | 19 |
|  |  | +Ve | 16 | 21 | 27 | 10 | 16 | 18 | 33 | 4 | 21 | 16 |

**Table S3.** Chi-square test for the blaKPC genotype against the four phenotypes among the 71 patients

|  |  | blaKPC | |  |
| --- | --- | --- | --- | --- |
|  |  | -Ve | +Ve | *Χ*^2^ |
| Carba Inactiv | -Ve | 9 | 16 | 0.500 |
|  | +Ve | 13 | 33 |  |
| MHT | -Ve | 5 | 20 | 0.096 |
|  | +Ve | 17 | 26 |  |
| Blue Carba | -Ve | 5 | 4 | 0.088 |
|  | +Ve | 17 | 45 |  |
| EDTA | -Ve | 6 | 28 | .020* |
|  | +Ve | 16 | 21 |  |

**Table S4**. Chi-square test for the blaNDM genotype against the four phenotypes among the 71 patients

|  |  | blaNDM | |  |
| --- | --- | --- | --- | --- |
|  |  | -Ve | +Ve | *Χ*^2^ |
| Carba Inactiv | -Ve | 22 | 3 | 0.684 |
|  | +Ve | 38 | 7 |  |
| MHT | -Ve | 25 | 0 | .009* |
|  | +Ve | 33 | 10 |  |
| Blue Carba | -Ve | 7 | 2 | 0.464 |
|  | +Ve | 53 | 8 |  |
| EDTA | -Ve | 33 | 0 | .001* |
|  | +Ve | 27 | 10 |  |

**Table S5.** Chi-square test for the blaVIM genotype against the four phenotypes among the 71 patients

|  |  | blaVIM | |  |
| --- | --- | --- | --- | --- |
|  |  | -Ve | +Ve | Chi-Square |
| Carba Inactiv | -Ve | 14 | 9 | 0.350 |
|  | +Ve | 31 | 12 |  |
| MHT | -Ve | 23 | 1 | .000* |
|  | +Ve | 20 | 21 |  |
| Blue Carba | -Ve | 6 | 3 | 0.943 |
|  | +Ve | 38 | 18 |  |
| EDTA | -Ve | 28 | 3 | .000* |
|  | +Ve | 16 | 18 |  |

**Table S6.** Chi-square test for the blaGIM genotype against the four phenotypes among the 71 patients

|  |  | blaGIM | |  |
| --- | --- | --- | --- | --- |
|  |  | -Ve | +Ve | Chi-Square |
| Carba Inactiv | -Ve | 23 | 2 | 0.523 |
|  | +Ve | 44 | 2 |  |
| MHT | -Ve | 24 | 1 | 0.615 |
|  | +Ve | 40 | 3 |  |
| Blue Carba | -Ve | 8 | 1 | 0.446 |
|  | +Ve | 59 | 3 |  |
| EDTA | -Ve | 34 | 0 | 0.048* |
|  | +Ve | 33 | 4 |  |

**Table S7.** Chi-square test for the blaOXA 48 genotype against the four phenotypes among the 71 patients

|  |  | blaOXA 48 | |  |
| --- | --- | --- | --- | --- |
|  |  | -Ve | +Ve | Chi-Square |
| Carba Inactiv | -Ve | 16 | 9 | 0.099 |
|  | +Ve | 20 | 26 |  |
| MHT | -Ve | 16 | 9 | 0.115 |
|  | +Ve | 19 | 24 |  |
| Blue Carba | -Ve | 6 | 3 | 0.305 |
|  | +Ve | 30 | 32 |  |
| EDTA | -Ve | 15 | 19 | 0.287 |
|  | +Ve | 21 | 16 |  |

**Table S8.** Spearman significant correlations among all possible genotypes and phenotypes combinations.

| Sample 1 | Sample 2 | | Correlation | | 95% CI for ρ | | P-Value |  |
| --- | --- | --- | --- | --- | --- | --- | --- | --- |
| blaVIM + blaGIM + blaNDM | MHT + Blue Carba + EDTA | | 0.774 | | (0.640, 0.862) | | 0 |  |
| blaVIM + blaNDM | MHT + EDTA | | 0.763 | | (0.625, 0.855) | | 0 |  |
| blaVIM + blaGIM + blaNDM | EDTA + Carba Inactiv + MHT | | 0.736 | | (0.587, 0.836) | | 0 |  |
| blaVIM + blaNDM | EDTA | | 0.724 | | (0.571, 0.828) | | 0 |  |
| blaVIM + blaNDM | MHT + Blue Carba + EDTA | | 0.718 | | (0.563, 0.824) | | 0 |  |
| blaVIM + blaGIM + blaNDM | MHT | | 0.711 | | (0.548, 0.821) | | 0 |  |
| blaVIM + blaNDM | EDTA + Carba Inactiv + MHT | | 0.683 | | (0.516, 0.800) | | 0 |  |
| blaVIM + blaNDM | MHT | | 0.679 | | (0.507, 0.800) | | 0 |  |
| blaVIM + blaGIM + blaNDM | Blue Carba + EDTA | | 0.678 | | (0.509, 0.797) | | 0 |  |
| blaVIM + blaGIM + blaNDM | Number of phenotypes | | 0.653 | | (0.477, 0.779) | | 0 |  |
| blaVIM + blaGIM + blaNDM + blaO | MHT | | 0.653 | | (0.473, 0.781) | | 0 |  |
| blaVIM + blaNDM | Number of phenotypes | | 0.623 | | (0.438, 0.757) | | 0 |  |
| blaVIM + blaGIM + blaNDM + blaO | MHT + EDTA | | 0.617 | | (0.431, 0.753) | | 0 |  |
| blaVIM + blaGIM + blaNDM + blaO | EDTA + Carba Inactiv + MHT | | 0.61 | | (0.421, 0.747) | | 0 |  |
| blaVIM + blaNDM | Blue Carba + EDTA | | 0.607 | | (0.418, 0.746) | | 0 |  |
| blaVIM + blaNDM + blaOXA-48 | MHT | | 0.6 | | (0.404, 0.743) | | 0 |  |
| blaVIM + blaGIM + blaNDM + blaO | MHT + Blue Carba + EDTA | | 0.593 | | (0.401, 0.735) | | 0 |  |
| blaVIM + blaGIM | MHT + EDTA | | 0.581 | | (0.386, 0.726) | | 0 |  |
| blaVIM + blaGIM + blaNDM | Carba Inactiv + EDTA | | 0.581 | | (0.386, 0.726) | | 0 |  |
| blaVIM + blaGIM | EDTA | | 0.577 | | (0.381, 0.723) | | 0 |  |
| blaVIM + blaNDM | MHT + Blue Carba | | 0.559 | | (0.359, 0.710) | | 0 |  |
| blaVIM + blaGIM + blaNDM | MHT + Blue Carba | | 0.558 | | (0.358, 0.709) | | 0 |  |
| blaVIM + blaGIM + blaNDM + blaO | Number of phenotypes | | 0.558 | | (0.358, 0.709) | | 0 |  |
| blaVIM + blaGIM | MHT + Blue Carba + EDTA | | 0.548 | | (0.346, 0.702) | | 0 |  |
| blaVIM + blaGIM + blaNDM + blaO | MHT + Blue Carba | | 0.544 | | (0.341, 0.698) | | 0 |  |
| blaVIM + blaNDM + blaOXA-48 | EDTA + Carba Inactiv + MHT | | 0.541 | | (0.338, 0.697) | | 0 |  |
| blaVIM + blaNDM + blaOXA-48 | MHT + EDTA | | 0.533 | | (0.328, 0.690) | | 0 |  |
| blaVIM + blaNDM + blaOXA-48 | MHT + Blue Carba + EDTA | | 0.524 | | (0.317, 0.683) | | 0 |  |
| blaVIM + blaNDM | Carba Inactiv + EDTA | | 0.523 | | (0.316, 0.682) | | 0 |  |
| blaVIM + blaNDM + blaOXA-48 | MHT + Blue Carba | | 0.518 | | (0.310, 0.679) | | 0 |  |
| blaVIM | MHT + EDTA | | 0.517 | | (0.307, 0.679) | | 0 |  |
| blaVIM + blaGIM + blaNDM + blaO | Carba Inactiv + MHT | | 0.515 | | (0.306, 0.676) | | 0 |  |
| blaVIM + blaGIM + blaNDM + blaO | EDTA | | 0.515 | | (0.307, 0.677) | | 0 |  |
| blaVIM + blaNDM + blaOXA-48 | | Number of phenotypes | | 0.509 | | (0.299, 0.672) | | 0 |
| blaVIM | | MHT + Blue Carba + EDTA | | 0.502 | | (0.290, 0.668) | | 0 |

**Figure S1**: Flow chart for detection of CPO in resource-limited settings
